# SPECT/CT bone scintigraphy to evaluate low back pain in young athletes: common and uncommon etiologies

**DOI:** 10.1186/s13018-016-0402-1

**Published:** 2016-07-07

**Authors:** M. Matesan, F. Behnia, M. Bermo, H. Vesselle

**Affiliations:** Nuclear Medicine, Department of Radiology, University of Washington, 1959 NE Pacific Street, Box 357115, Seattle, 98195-0001 USA

**Keywords:** SPECT/CT, Back pain, Young athletes, Referred pain

## Abstract

Low back pain of various etiologies is a common clinical presentation in young athletes. In this article, we discuss the utility of SPECT/CT bone scintigraphy for the evaluation of low back pain in young athletes. The spectrum of lower spine lesions caused by sports injuries and identifiable on bone scan is presented along with strategies to avoid unnecessary irradiation of young patients. Also covered are pitfalls in diagnosis due to referred-pain phenomenon and normal skeletal variants specific to this age group.

## Background

The etiology of low back pain in young athletes differs from that seen in adults, with bony etiology being more common than disc-related disease [1,2]. Chronic low back pain occurs more often than acute pain and is caused by repetitive microtrauma due to flexion, extension, and rotation movements that increase the risk of injury to the posterior elements of the spine.

The initial evaluation of a young adult with back pain starts with lumbar spine radiograph; however this has limited sensitivity in the detection of pars fractures and stress reactions. MRI is preferred if neurologic symptoms are present or if radicular pain is identified on clinical exam. In the absence of neurologic symptoms, evaluation can proceed by bone scintigraphy with planar and SPECT acquisitions. Bone scintigraphy can differentiate acute spondylolysis from old chronic nonunion fracture, and there is a good correlation between a positive bone scan and painful pars lesion [3]. Bone scan with SPECT is superior to MRI and CT in the detection of spondylolysis [2]. One study comparing SPECT and CT versus MRI showed that only 40 out of 50 lesions seen on SPECT were revealed by MRI and there was no case of positive MRI with negative SPECT [4].

Besides evaluation for marrow edema at the pars interarticularis (high signal intensity on STIR images and low signal in T1), MRI offers a good evaluation of surrounding soft tissue lesion (like posterior ligamentous complex), spinal cord, and intervertebral disc.

18F NaF PET scan has been also shown to be useful in the detection of a variety of skeletal abnormalities in young patients with back pain with higher resolution and similar radiation dosimetry but a higher cost relative to Tc99m-MDP [5].

SPECT images have better contrast resolution compared to planar images and detect additional sites of abnormal uptake, in one study performed in patients with low back pain in 24 % of the cases [6]. The large field-of-view surveyed by planar whole-body imaging offers the advantage of identifying additional abnormal bony sites that may trigger referred pain or mimic radicular pain unsuspected upon initial clinical evaluation.

Referred pain in the lower lumbar region originating from the sacroiliac joint and hip can be explained by the common innervation of the hip/sacroiliac joints and intervertebral discs by sacral and lower lumbar nerves [7,8]. Studies have shown that patients with low back pain, with or without leg pain, may have the spine, sacrum/sacroiliac joints, or hip as the cause of their symptoms [7–9]. A study published by Sembrano et al. in patients with low back pain, with or without leg pain, showed that in 65 % of cases, the major pain generator came from the spine only; in 5 %, it originated from the sacroiliac joint only; and in 2.5 % of the cases, the back pain originated from the hip only [8]. Because attention is directed to the referral site of pain, the area that represents the source of pain may be overlooked if the imaging field of view is limited. Therefore, correlation with clinical history and physical exam and consideration of the risk factors for a certain pathology type are important in avoiding a delay in diagnosis. For example, long distance female runners are at slightly higher risk for sacral stress fractures that clinically may manifest as low back pain and buttock pain mimicking radicular pain [10]. Although spinal radicular pain has certain characteristics (dermatomal distribution, extension beyond the knee, and sensory or motor loss), there is a high incidence of nonradicular pain mimicking radicular pain [9].

### Bone scan protocol

Planar (whole body or spot) scintigraphic images are obtained 3–4 h after intravenous injection of 9.3 MBq/kg (0.25 mCi/kg) of Tc99m-MDP (methylene diphosphonate), followed by SPECT acquisition with or without additional CT acquisition [11]. Blood flow and blood pool images may be added if an acute injury is suspected. Planar images include a large area encompassing more than the site of pain to cover areas of potential referred pain.

A standard SPECT acquisition protocol for bone imaging with a dual-head gamma camera consists of 25 s per view, 60 view angles over 180° (3° increments between views), and 128 × 128 binning.

Recent SPECT-CT systems are equipped with fully diagnostic quality CT systems; however, usually, only low-dose CT is obtained for attenuation correction and for visualization of bony details over a limited field of view thereby minimizing the impact of radiation on the patient’s gonads. Our routine low-dose CT consists of a 60-mA tube current and a 0.8-s tube rotation at 120 kVp. In the absence of any abnormality on SPECT images, the CT is not performed, thereby sparing young patients the additional radiation. Rarely, a diagnostic beam CT over a limited filed of view is required for anatomic characterization.

### Lumbar spine origin of low back pain

#### Spondylolysis

Isthmic spondylolysis represents a pars interarticularis fracture usually associated with repetitive forced hyperextension and rotation. It has a higher incidence during the adolescent growth spurt due to incomplete bone maturation of the neural arch and repetitive stress injury [1,2] but heredity also plays a role [12]. It is common for spondylolysis to be bilateral, and the vast majority of cases occur at L5 with the next more common location being L4 [13]. Spondylolysis usually presents with focal chronic low back pain which is unilateral or bilateral and increases with activity, without radicular symptoms.

**Fig. 1:**
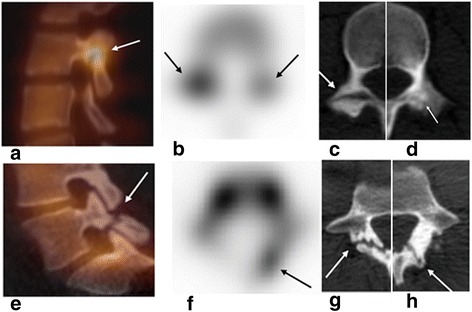
Isthmic spondylolysis: progressive lesion versus terminal stage of chronic nonunited lesion. **a** Sagittal SPECT/CT image in a 16-year-old female gymnast complaining of lumbar back pain showed intense increased uptake at the right L3 pars defect. Uptake at bilateral L3 pars fractures more prominent on the right is seen in axial SPECT image (**b**) suggesting progressive lesions more acute on the right; axial CT images at two slightly different levels of L3 vertebra (**c**, **d**) show bilateral fracture line defects. **e** Sagittal SPECT/CT image and axial SPECT image (**f**) in a 19-year-old female with remote history of cheerleading, presenting with low back pain showed no uptake on the chronic nonunited right L5 pars defect; axial CT images at two slightly different levels of L5 vertebra (**g**, **h**) showed fracture line and sclerosis on the right and sclerosis, overgrowth of the contralateral (*left*) posterior elements and lamina fracture due to stress and bony remodeling in an unstable posterior neural arch

Detection of a pars defect by lumbar spine radiograph still needs further evaluation with SPECT/CT or MRI to confirm active remodeling at the site of pars defect (increased radiotracer uptake or marrow edema respectively). The spectrum of lesions at the pars interarticularis includes stress reaction without spondylolysis (increased uptake on planar images and/or SPECT without associated abnormality or bony sclerosis without fracture line on CT), spondylolysis (increased uptake on SPECT and fracture line on CT), and old healed nonunion (no increased bone uptake and a fracture line with sclerotic margins on CT). Unilateral spondylolysis may be accompanied by contralateral stress demonstrated as increased bone uptake on SPECT and sclerosis on CT in the contralateral pars interarticularis. Advanced cases may present with contralateral sclerosis and overgrowth of the pedicle and lamina due to compensatory physiologic response in an unstable neural arch [14,15]. Examples of spondylolysis lesions are presented in Fig. 1.

### Lumbar spine pathology other than spondylolysis

Although isthmic spondylolysis is the most common cause of low back pain in young athletes, other etiologies need to be considered. These include stress reaction or fracture at the pedicle, transitional vertebra, lumbar interspinous bursitis, traction apophysitis (at the iliac crest, spinous process, or anterior vertebral ring apophysis), facet joint disease, facet posterior fracture in the lumbar spine region, avulsion fracture of the secondary ossification centers, endplate degenerative changes, and sacral facet fracture. Ligament injuries cannot be diagnosed on bone scan unless resulting in calcification.

#### Pedicle stress reaction or fracture

**Fig. 2:**
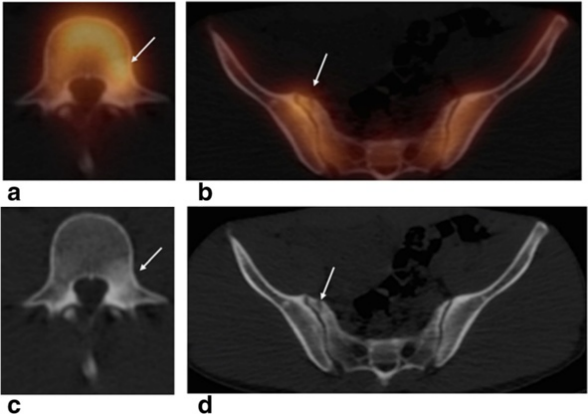
Pedicle stress reaction in an 18-year-old male wrestler complaining of left-sided back pain. Incidental finding of a small old avulsion fracture of the right sacral ala at the joint capsule insertion **a** Axial fused SPECT/CT image shows mildly increased uptake at the left L4 pedicle, which on axial CT image (**c**) is associated with sclerosis in the left pedicle region. **b** Fused SPECT/CT axial image of the sacrum shows minimal uptake associated with a small avulsion fracture at the right sacral ala on axial CT image (**d**)

The pedicle is the second weakest point in the neural arch. Injuries to the pedicle are commonly seen in association with contralateral (unilateral) spondylolysis; however, isolated injuries have been also reported [16]. Focal uptake on bone scan helps in differentiating between a stress fracture in this region and an old defect for example due to a developmental retrosomal (pedicular) cleft in this region [17]. One example of pedicle stress reaction is shown in Fig. 2.

#### Fractures at the vertebral apophyses

In the adolescent age range, fractures at the secondary centers of ossification of the vertebrae (apophyses) can occur since most of them are not yet fused. There are seven secondary centers of ossification (apophyses) in a lumbar vertebra as described below. On bone scan, only faint uptake is usually seen associated with these apophyses and high and asymmetric uptake at one of the secondary ossification centers is suggestive of an avulsion fracture.

#### Limbus vertebra

Injury to the ring apophysis caused by disc material protruding through the growth plate of ring apophysis can result in limbus vertebra. This is seen as a small corticated bony fragment matching osseous defect at the superior margin usually in anterosuperior location and on imaging that needs to be differentiated from an acute vertebral fracture, a Schmorl nodule, or calcified disc herniation. Limbus vertebra is usually asymptomatic unless it has a posterior location when it can potentially cause a neurologic symptom [18].

#### Fractures of the transverse processes of the lumbar vertebrae

Fractures of the transverse processes of the lumbar vertebrae may result from violent lateral flexion-extension forces (as in football) [19]. Fractures of the L5 transverse process raise the suspicion for sacral fracture.

#### Lumbosacral transitional vertebrae syndrome (Bertolotti syndrome)

**Fig. 3:**
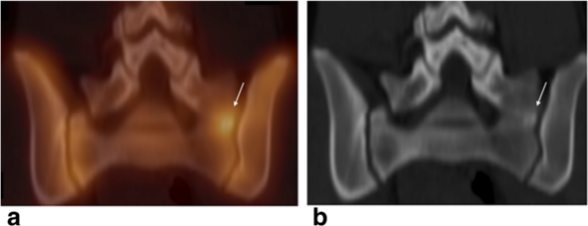
Lumbarization of the S1 vertebra with anomalous articulation of the left side of S1 to the remainder of the sacrum and with the left iliac bone in a 16-year-old female gymnast with left-sided low back pain. **a** Coronal fused SPECT/CT image shows intense uptake at the left S1–S2 pseudoarthrosis. **b** Coronal CT image shows lumbarization of S1 with articulation with S2 instead of fusion, with marginal sclerosis seen on the *left side* (*arrow*) likely due to degenerative changes

Lumbosacral transitional vertebrae syndrome (Bertolotti syndrome) is defined as either sacralization of the lowest lumbar segment or lumbarization of the most superior sacral segment of the spine. It is present in 3–21 % of the population [20]. The sacralization of the fifth lumbar vertebra into the sacrum occurs in 6 % of American adults, and the vertebra can fuse at one or more locations (between the transverse process, vertebral body, or facet joints) [21]. Pseudoarthrosis can undergo degenerative changes or may increase stress to the joint space above the transitional vertebrae or to the contralateral facet [20] mainly in sports with repeated hyperextension movements. Abnormal articulation with associated altered weight-bearing biomechanics can predispose to unusual fracture patterns that might be hard to differentiate from anomalous articulation either on CT or on SPECT due to associated bone remodeling in both conditions. On CT, stress reaction at this articulation may be seen as marginal spurring, hypertrophy, and sclerosis [22]. Figure 3 shows an example of increased uptake associated with pseudoarticulation at a transitional vertebra.

#### Impingement syndrome of the adjacent spinous processes (Baastrup disease-lumbar interspinous bursitis)

Impingement syndrome of the adjacent spinous processes (Baastrup disease-lumbar interspinous bursitis) can be due to a tight thoracolumbar fascia with accentuated lordosis and worsened by excessive hyperextension and hyperflexion [23].

#### Facet joint

Facet joint can be also a source of pain if degenerative changes are present and shows increased uptake on bone scan.

#### Adolescent disc dysplasia

**Fig. 4:**
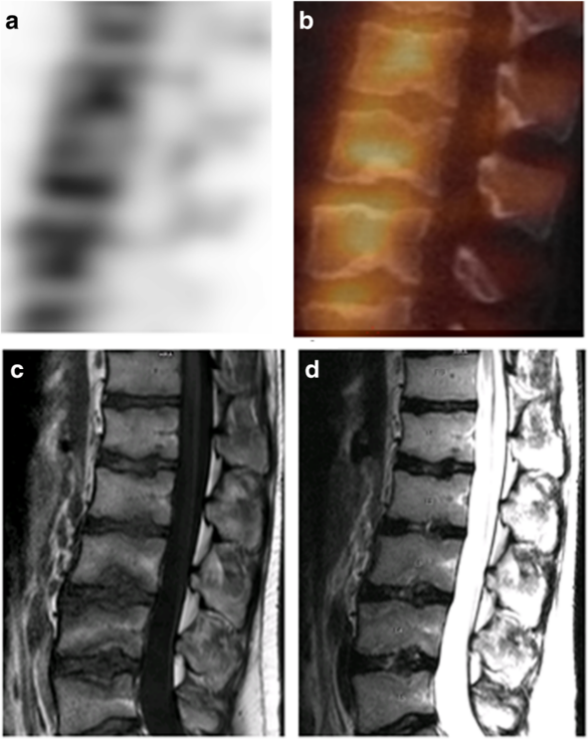
Twenty-year-old male with chronic longstanding lumbar pain with physical activities due to adolescent disk dysplasia. Bone scan shows heterogeneous uptake in the spine associated with endplates changes. Sagittal bone SPECT image of the lumbar spine (**a**) and fused SPECT/CT images (**b**) show heterogeneity of uptake in the vertebral bodies. Sagittal T1 (**c**) and T2 (**d**) weighted MR sequences showed straightening of the lumbar lordosis, endplate disc desiccation seen from T12-L1 through L5-S1, Schmorl's nodes at multiple levels, and early Modic type 1 endplate degenerative changes.

Significant chronic back pain in the thoracic or lumbar spine without spinal deformity associated with disc space narrowing, endplate irregularity, and Schmorl’s nodes has been defined as adolescent disc dysplasia [24] (Fig. 4).

### Referred lower lumbar pain

Referred lower lumbar pain can be triggered by pathologic processes localized in the sacrum (sacral fractures), sacroiliac joints (sacroiliac joint syndrome), or hips [8,9]. The large field of view of planar bone scan helps in identifying these possible etiologies of lower lumbar pain.

#### Sacral stress fracture

**Fig. 5:**
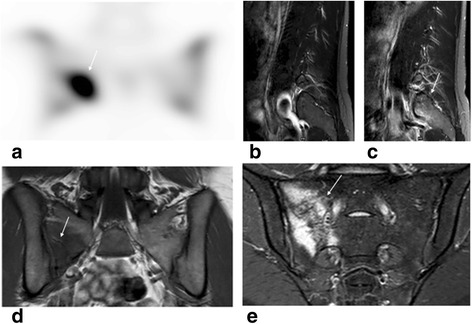
Twenty-two-year-old female long distance runner with sacral stress fracture which initially mimicked radicular pain (lower lumbar pain with pain/numbness in buttock) and later the pain was localized over the right sacroiliac joint. **a** Coronal SPECT image of the pelvic region shows intense uptake in the right lateral aspect of the sacrum. **b**, **c** Sagittal T2 STIR MR sequences *left* and *right*, respectively, performed 1 month prior to bone scan for “radicular type lumbar pain” was read as negative, but coronal pelvic T2-weighted images were not obtained. In retrospect sagittal T2 STIR image right (**c**) showed edema in the right sacrum (seen at the edge of the field of view only). Repeat MRI of the pelvis 1 day after bone scan showed nondisplaced right lateral sacral fracture on coronal T1 weighted images (**d**) with associated bone marrow edema seen in coronal T2-weighted SPAIR image (**e**). Athletes with lower lumbar pain and negative lumbar spine MRI would benefit from additional coronal pelvis images to exclude referred lumbar pain caused by pelvic injuries

Stress fractures of the pelvis are seen mainly in female athletes who are long distance runners. A superimposed decreased bone density is a predisposing factor in young athletes with the “female athlete triad” (decreased bone mineral density, amenorrhea, poor nutrition). Pain is localized to the lumbar or buttock region with extension to the spine, but the pain can also mimic sciatica due to disc disease [25]. Since sacral fracture might be missed on routine protocol lumbosacral MRI, it is helpful to add coronal STIR acquisition of the sacrum in cases where this is clinically suspected (Fig. 5).

#### Sacroiliac joint syndrome

Sacroiliac joint syndrome is due to increased sports activities with increased abnormal motion or stress at the sacroiliac joint. This can be difficult to diagnose on bone scintigraphy given the normally increased uptake in this age group patient population [26]. Degenerative changes can be seen on CT: sacroiliac joint irregularity, subchondral cysts, and sclerosis [27].

### Low back pain in young athletes not related to sports activity

**Fig. 6:**
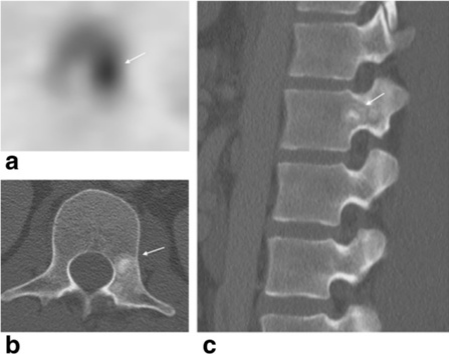
Osteoid osteoma lesion at L1 pedicle with “double intensity sign” on bone scan. **a** Axial SPECT image show focal increased uptake in the left pedicle with a surrounding area of less intense uptake (“double intensity sign“). **b** Axial CT image of L1 vertebra and **c** sagittal image of the spine (between T12 and L3 levels) show a sclerotic lesion at L1 left pedicle with a central lucency which is almost completely calcified

Since bone scintigraphy has limited specificity, correlation with additional imaging, particularly with CT is necessary to further clarify the bony abnormality. Differential diagnosis for uptake in a vertebra in this age group includes infection, benign lesions like osteoid osteoma, osteoblastoma, aneurysmal bone cyst, and chondroblastoma, and rare malignant lesions like osteosarcoma. Osteoid osteoma and osteoblastoma often involve the posterior spinal elements, with the thoracolumbar spine being the most common site of involvement. Osteoid osteoma affects the spine in 10–20 % of cases [28] and is presenting as a sclerotic lesion with a radiolucent nidus (Fig. 6). Patients have back pain, spinal deformity, and nerve root compression symptoms. The most common location is in the posterior elements lamina, facets, pedicle, pars interarticularis, and spinous process. Osteoid osteoma on bone scan appears as a focus of increased radiotracer uptake occasionally with a surrounding area of less intense uptake (“double intensity sign”) [29]. Chondroblastoma is a benign but aggressive neoplasm that may also occur in the vertebral column (vertebral bodies and posterior elements). Osteoblastoma and chondroblastoma occasionally may be associated with an aneurysmal bone cyst.

Discitis or osteomyelitis can also present with increased uptake on bone scan. In case of discitis, there is radiotracer uptake along the affected facing endplates.

### Pitfalls

In the first few hours after acute trauma, bone scintigraphy may be falsely negative; however, sensitivity is closed to 100 % at 72 h after fracture onset [30].

Also, it is important to be familiar with normal skeletal development across different age groups. There are 3 primary ossification centers for a spinal vertebra and a total of 21 for sacrum, and these fuse before puberty (usually by 6–7 years old) and are normally not seen any more in the adolescent age range. However, recognizing confounding variants due to failure of the segmentation process or fusion abnormalities of these centers is important [15,31].

**Fig. 7:**
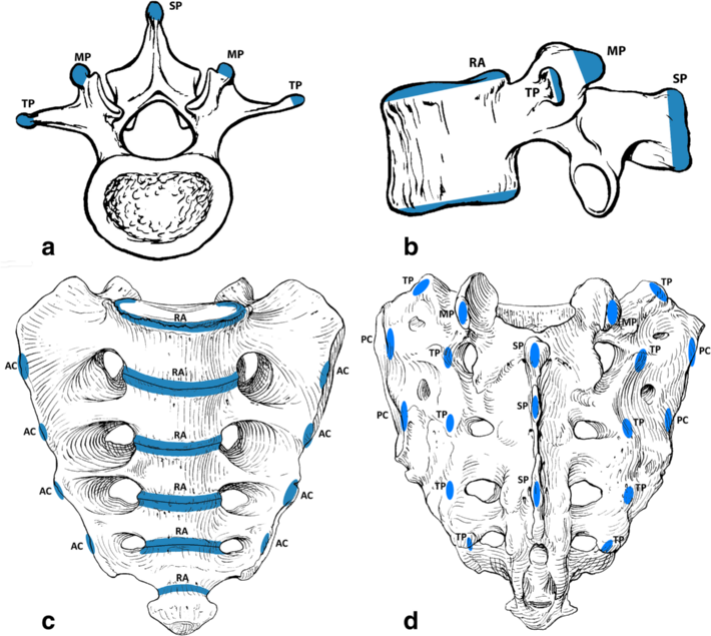
Expected location of the secondary ossification centers in the lumbar vertebra and sacrum. Schematic illustration of a lumbar vertebra; axial view (**a**) and lateral view (**b**) shows secondary ossification centers at the spinous process (*SP*), transverse process (*TP*), mammillary process (*MP*), and the superior and inferior ring apophysis (*RA*) that encircle the developing endplate. Schematic illustration of the sacrum anterior (**c**) and posterior view (**d**) shows secondary ossification centers at the transverse process apophysis (TPA), anterior costal process apophysis (ACA), posterior costal process apophysis (PCA), mammillary process apophysis (MPA), spinous process apophysis (SPA), and the ring apophyses (RA) [32]. Copyright: LifeArt from photosearch.com

In adolescence, the secondary ossification centers start to be seen. There are seven apophyses for each lumbar vertebra, five for a thoracic vertebra, and a variable number for the sacrum. They develop during childhood and fuse during late adolescence and early adult life. Expected location for these ossification centers is shown in Fig. 7 [32–34].

**Fig. 8:**
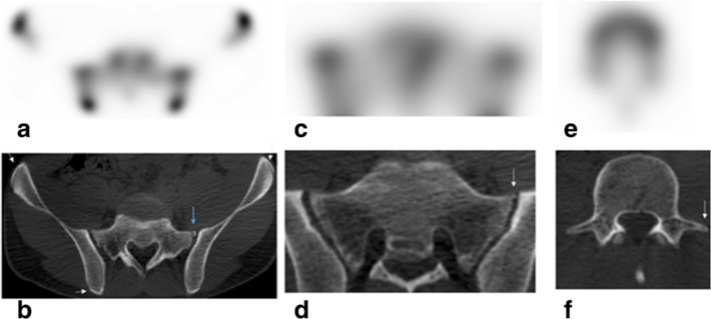
Normal appearance of bone scan and CT scan in a 17-year-old male with mild symmetric uptake in the secondary ossification centers (apophyses). Normal prominent uptake is seen associated with iliac crest apophysis (secondary ossification center) which starts ossifying in this age range and fuses later (until 25 years old). **a** Axial SPECT image shows symmetric uptake seen in the iliac crest apophyses and subtle uptake associated with small left S1 transverse process apophysis (*blue arrow*) seen on the corresponding CT (**b**). **c** Axial SPECT image shows no significantly increased uptake in the small anterior costal epiphysis seen on axial CT image in the expected location (arrow in **d**). **e** Axial SPECT image of a lumbar vertebra with no significant uptake seen on axial CT image at the transverse process apophysis on the left (*arrow* on **f**). Familiarization with the normal appearance and location of the secondary ossification centers is important in the interpretation of back pain to avoid confusion with a small avulsion fractures

These ossification centers have mild but usually symmetric radiotracer uptake; when fusion of the ossification centers is asymmetric, it can be confused with a fracture [34]. Minimal Tc99m-MDP uptake not higher than other ossification centers with smooth round well-corticated margins on CT and expected location may help to differentiate a secondary ossification center from a small fracture (Fig. 8) [32]. However, intense uptake in an apophysis may represent acute avulsion fracture at that level. Confounding variants can be an anomalous S1/S2 articulation, nonunited ossification center of the superior articular facet at S1, normal variance in appearance of the first sacral segment, unilateral sacral rib, and unfused ossicle (more common at the inferior articular process L2 or L3) [32,34,35].

## Conclusions

Bone scan is a useful clinical tool to explore the etiology of low back pain like spondylolysis and other less common etiologies in young athletes. It is also particularly important to detect the active source of pain when more than one bony abnormality is seen in anatomical imaging. The addition of SPECT-CT increases the clinical accuracy due to increased contrast resolution and anatomical localization.
